# Recent Progress in “Water-in-Salt” Electrolytes Toward Non-lithium Based Rechargeable Batteries

**DOI:** 10.3389/fchem.2020.00595

**Published:** 2020-08-07

**Authors:** Yuyan Wang, Xiaotang Meng, Jinfeng Sun, Yang Liu, Linrui Hou

**Affiliations:** School of Materials Science & Engineering, University of Jinan, Jinan, China

**Keywords:** “water-in-salt” electrolytes, aqueous batteries, non-lithium, rechargeable metal ion batteries, high energy density

## Abstract

Aqueous non-lithium based rechargeable batteries are emerging as promising energy storage devices thanks to their attractive rate capacities, long-cycle life, high safety, low cost, environmental-friendliness, and easy assembly conditions. However, the aqueous electrolytes with high ionic conductivity are always restricted by their intrinsically narrow electrochemical window. Encouragingly, the highly concentrated “water-in-salt” (WIS) electrolytes can efficiently expand the stable operation window, which brings up a series of aqueous high-voltage rechargeable batteries. In the mini review, we summarize the latest progress and contributions of various aqueous electrolytes for non-lithium (Na^+^, K^+^, Zn^2+^, Mg^2+^, and Al^3+^) based rechargeable batteries, and give a brief exploration of the operating mechanisms of WIS electrolytes in expanding electrochemically stable windows. Challenges and prospects are also proposed for WIS electrolytes toward aqueous non-lithium rechargeable metal ion batteries.

## Introduction

Recently, the safety issues and production costs of rechargeable batteries become the main factors restricting their commercial applications in portable electronic devices (PED), electrical vehicles (EV), and stationary electronic energy storage systems (EESs) (Wang et al., 2007; Suo et al., 2015; Lukatskaya et al., 2018). Thus, how to effectively select appropriate materials involved in batteries has become an important and challenging topic. As an important component of batteries, the electrolytes play a vital role in the superior electrochemical performance of batteries, and have attracted more and more attention in recent years (Kandhasamy et al., 2012; Yan et al., 2012; Suo et al., 2013). Although traditional organic electrolytes have exhibited appealing applications in rechargeable batteries, they inherently contain a large amount of expensive yet flammable organic solvents with certain levels of toxicity, which makes the device assembly conditions relatively harsh (Wang et al., 2012; Xu and Wang, 2016; Yang et al., 2019a). Consequently, aqueous electrolytes have been established as promising alternative candidates for advanced rechargeable batteries since their first application in lithium ion batteries (LIBs) (Li et al., 1994). The aqueous batteries display distinct merits, including low cost, high safety, high electronic conductivity, mild assembly environment, and so on (Wang et al., 2007; Baskar et al., 2014; Huang et al., 2019a). However, the electrochemical stability voltage window of aqueous batteries is as narrow as ~1.23 V, which seriously restricts the optimal choice of cathode and anode materials due to the existence of hydrogen and/or oxygen evolution reactions. This excludes most electrochemical couples that occur above the output voltage of 1.5 V, which limits the enhancement in energy density of full devices (Lu et al., 2011; Kim et al., 2014; Jiang et al., 2019a; Liu et al., 2020).

Recently, highly concentrated “water-in-salt” (WIS) electrolytes, in which the dissolved salts far outnumbers the water molecules (salt/solvent ratio > 1 by volume or weight), have been reported to expand the stable voltage window up to ~3.0 V (Suo et al., 2016, 2017). In the WIS electrolytes, all water molecules participate in the ion solvation shells, and no “free” water remainders can be found. As a typical system, Suo et al. first developed a high concentration electrolyte with 21 m (mol kg^−1^) of lithium bis(trifluoromethylsulfonyl)imide (LiTFSI) for aqueous rechargeable LIBs (the molar ratio of Li^+^ to H_2_O is 2.5) (Wang et al., 2015). However, the economic and environmental concerns, as well as the rarity and increasing consumption of Li resources, restrain the scalable applications of lithium-based electrochemical devices. As a consequence, the development of alternative aqueous rechargeable batteries based on some other earth-abundant elements turns out to be urgent and more meaningful. Therefore, the monovalent (Na^+^, K^+^) and/or multivalent (Zn^2+^, Mg^2+^, and Al^3+^) cation based aqueous secondary batteries have been intensively explored recently (Wessells et al., 2011; Zhao et al., 2016; Suo et al., 2017; Wang et al., 2020).

In this mini review, we mainly addressed the topic of the WIS electrolytes and their latest progress in various non-lithium aqueous rechargeable metal-ion batteries (ARMIBs). In the first section, we gave a brief exploration of the involved mechanism of WIS electrolytes in extending the electrochemical stability voltage window of devices. And then, we provided an extensive overview of the applications of WIS electrolytes in aqueous non-lithium secondary batteries, including aqueous sodium-ion batteries (ASIBs), aqueous potassium-ion batteries (APIBs), aqueous zinc-ion batteries (AZIBs), aqueous magnesium-ion batteries (AMIBs), and aqueous aluminum-ion batteries (AAIBs). Finally, we proposed the existing challenges and prospects for the future development of WIS electrolytes toward advanced non-lithium ARMIBs.

## The Operating Mechanism of “WIS” Electrolytes in Extending the Electrochemical Window of Devices

It is well-known that free water fraction is one of the key factors affecting the electrochemical stability of electrolytes (McEldrew et al., 2018; Vatamanu and Borodin, 2018). In traditional “salt-in-water” (SIW) electrolytes, the water molecules enormously outnumber the salts, and are relatively free to form hydrogen bonding networks. Thus, a large amount of water molecules will separate or solubilize (or corrode) the electrode material (Dubouis et al., 2018; Huang et al., 2019a). With the salt concentration increasing, the tighter solvation shell associated with WIS electrolytes can be formed. Meanwhile, the “freedom” water solvent molecules display a lower mobility. They turn out to be preferentially solvated by metal ions, and thus less available to separate salt anions. Accordingly, the water-water hydrogen bonds are replaced by water-ion-bonding interactions, enhancing the interactions between cations and anions, which can further widen the stable working windows of electrolytes (Azov et al., 2018).

**Scheme 1:**
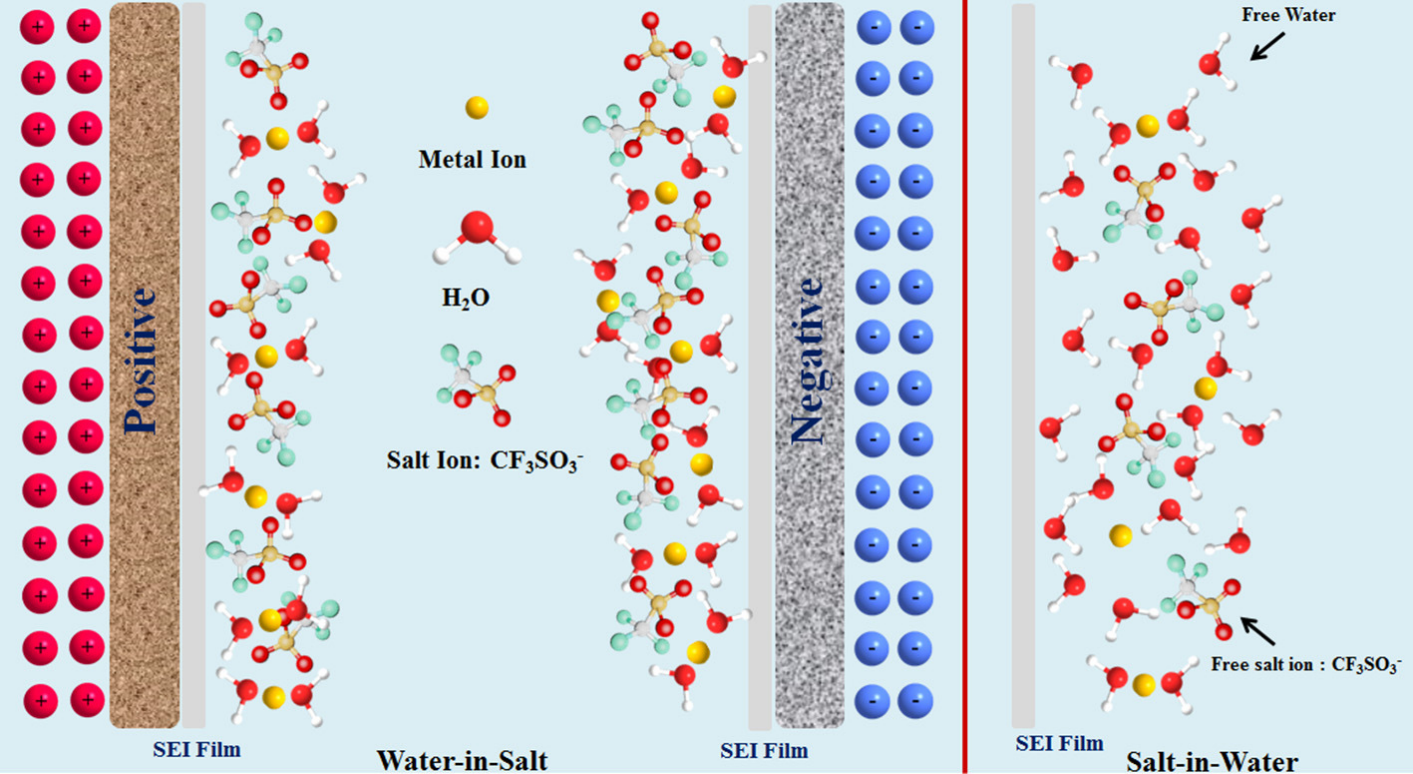
Schematic diagram of the solvation structure for WIS electrolytes. By Linrui Hou, et al.

It is also believed that the formation of a solid electrolyte interphase (SEI) layer with a high salt concentration on the electrode surface can prevent water reduction, thus positively contributing to the wide electrochemical stability window. To be specific, the OH^−^ generated during the hydrogen oxygen reaction in the first cycle will chemically react with anions (such as TFSI) to mainly form a stable SEI film, which further prevents water reduction, and enhances the oxidative stability of the electrode masteries (Coustan et al., 2018; Dubouis et al., 2018). A typical solvation structure for WIS electrolytes is schematically depicted in Scheme 1.

It is well-known that it is an effective strategy to increase the energy density of batteries by raising the operating voltage (Xia et al., 2017; Manalastas et al., 2018). The voltage, according to the following Nernst equation, is highly dependent upon the half-cell potentials of both positive and negative electrodes.

(1) $$\begin{array}{l} \Delta V=V_{+}-V_{-} \end{array}$$

(2) $$\begin{array}{l} V=V^{0}-\frac{RT}{nF}\ln\;\frac{C^{a_{red}}}{C^{b_{ox}}} \end{array}$$

where *V*^0^ is the standard half-cell reduction potential in respect to the standard hydrogen electrode (SHE), *R* is the universal gas constant, *T* is the absolute temperature in kelvins, *n* is the stoichiometric number of electrons, *F* is Faraday's constant, *C*_*red*_ is the concentration of the reduced form, *C*_*ox*_ is the concentration of the oxidized form, and *a* and *b* are exponential powers determined by the coefficients of *C*_*red*_ and *C*_*ox*_ in the redox half-reaction, respectively.

The maximum cell potential (Δ*V*) is critically determined by the potential subtraction between positive and negative (*V*^0^_+_-*V*^0^_−_). The higher concentration of the oxidized form (C_*ox*_) establishes stronger reduction potentials, as shown in Equations 1, 2. The electrolyte concentration and SEI film can effectively control the electrolyte decomposition, which allows even higher-concentration redox ions to participate in electrochemical reactions within the broadened electrochemical voltage window. Density functional theory (DFT) calculations are reported as an effective way to calculate reduction potentials of the anions' salts (Suo et al., 2015; Dawut et al., 2019; Shin et al., 2019).

## WIS Electrolytes for Non-Lithium Armibs

Considering some retrieved reviews about WIS electrolytes in lithium-based aqueous batteries (Wang et al., 2012; Kim et al., 2014), this mini-review will mainly focus on the latest progress in highly concentrated WIS electrolytes for non-lithium ARMIBs including ASIBs, APIBs, AZIBs, AMIBs, and AAIBs. The electrochemical properties of these batteries are systematically collected in Table 1.

**Table 1:** The main electrochemical properties for WIS-based aqueous non-lithium batteries.

**Metal**	**Radius (nm)**	**Electrolytes**	**Voltage (V)**	**electrodes**	**References**
Na	0.102	35 m NaFSI	2.6	NaTi_2_(PO_4_)_3_ anode Na_3_(VOPO_4_)_2_F cathode	Kühnel et al., 2017
		18.5 m Na(PTFSI)_0.65_(TFSI)_0.14_(OTf)_0.21_3H_2_O	2.7	Vs Na^+^/Na	Zheng et al., 2019
		Na(PTFSI)_0.65_(TFSI)_0.14_(OTf)_0.21_3H_2_O	1.75	Na_3_V_2_(PO_4_)_2_F_3_|NaTi_2_(PO_4_)_3_ full cell	
		17m NaClO_4_	2.8	Na_2_MnFe(CN)_6_| NaTi_2_(PO_4_)_3_	Nakamoto et al., 2017
		17m NaClO_4_	2.7	Vs Na^+^/Na	Lee et al., 2019
		17m NaClO_4_	1.8	Na_4_Fe_3_(PO_4_)_2_(P_2_O_7_)|NaTi_2_(PO_4_)_3_ full-cell	
		25m NaFSI + 10m NaTFSI	1.9	Na_3_(VOPO_4_)_2_F|NaTi_2_(PO_4_)_3_ full-cell	Reber et al., 2019
		9 m NaOTf + 22 m TEAOTf	3.3	Vs Ag/AgCl	Jiang et al., 2019a
K	0.138	27.7 m K(PTFSI)_0.12_(TFSI)_0.08_(OTf)_0.8_2H_2_O	2.5	vs K^+^/K	Zheng et al., 2019
		30 m KAc	3.2	KTi_2_(PO_4_)_3_ as anode	Leonard et al., 2018
		22 m KCF_3_SO_3_	3.0	vs Ag/AgCl	Jiang et al., 2019b
		22 m KCF_3_SO_3_	2.6	KFeMnHCF-3565|PTCDI full cells	
		30 m KFSI	3.97	vs. Hg/Hg_2_Cl_2_)	Chen et al., 2020
Zn	0.074	21 m LiTFSI +0.5 m ZnSO_4_	1.8	LiMn_0.8_Fe_0.2_PO_4_ as cathode	Zhao et al., 2016
		21 m LiTFSI+ 1 m Zn(CF_3_SO_3_)_2_	1.4	V_2_O_5_ as anode	Hu et al., 2017
		2.4 m KOH+1.0 m H_2_SO_4_ acid-alkaline dual electrolyte	2.44	Zn/Zn(OH)^2−^anode+ Mn^2+^/MnO_2_ as cathode	Liu et al., 2020
Al	0.0535	5 m Al(CF_3_SO_3_)_3_	2.65	PBA-type FeFe(CN)_6_ as cathode	Zhou et al., 2019
Mg	0.072	1 m MgSO_4_	1.5	PBN-Na_1.4_Ni_1.3_Fe(CN)_6_5H_2_O| polyimide full cells	Xia et al., 2017
		4 m Mg(TFSI)_2_	1.9	Li_3_V_2_(PO_4_)_3_|poly pyromellitic dianhydride	Wang et al., 2017

*FSI, bis(fluorosulfonyl)imide; TFSI, bis(trifluoromethylsulfonyl)imide; OTf, trifluoromethane sulfonate; PTFSI, N(SO_2_CF_3_)(SO_2_C_2_F_5_); TEA, tetraethylammonium; TFSI, bis(trifluoromethanesulfonyl)imide; PBN, Prussian blue; PTCDI, 3,4,9,10-perylenetetracarboxylic diimide*.

It is well-known that electrolytes as ionic transport intermediates, with their inherent ionic conductivity, mobility, interfacial characteristics, and other properties, play a critical role in enhancing the cycle performances. Designing and optimizing a functional electrolyte with a stable electrode/electrolyte interfaces has to be considered as an essential way to achieve a superior electrochemical performance in aqueous batteries. The typical design strategies are optimized by varying the electrolyte components, including salts and additives (Peng et al., 2017; Zhao et al., 2020).

The choice of salts significantly affects the electrochemical stability of the electrolytes, as well as their ionic conductivity and thermal stability. The salt anions in the aqueous WIS electrolytes can be commonly divided into inorganic (Cl^−^, ${\text{SO}}_{4}^{2-}$, and ${\text{ClO}}_{4}^{-}$) and organic (CF_3_${\text{SO}}_{3}^{-}$, FSI^−^, TFSI^−^, BETI^−^, and PTFSI^−^) ones (Hong et al., 2013; Zhang et al., 2020). Inorganic salts are likely to be considered due to their low cost and high ionic conductivity. For instance, the fewer side reactions and low oxidation of Cl^−^ make it suitable for aqueous electrolytes (Zhang et al., 2018). In the case of ${\text{SO}}_{4}^{2-}$, the low cost, good compatibility, and exceptional stability make it more attractive; however, some by-products produced by over cycling still limits its practical application (Zhao et al., 2016; Huang et al., 2019b). Another anion is ${\text{ClO}}_{4}^{-}$, which has strong oxidability, lowering the potential for explosive risks and high toxicity (Lee et al., 2019). The bulky organic anions (i.e., CF_3_${\text{SO}}_{3}^{-}$, FSI^−^, TFSI^−^, BETI^−^, and PTFSI^−^) in aqueous electrolytes can reduce the solvation effect by occupying a large space. These anions show low ionic conductivity and corrosion issues (Yamada et al., 2016; Jiang et al., 2019a; Pan et al., 2019).

### ASIBS

Sodium, as one an alkali metal, is closely located with lithium in the periodic table and has a relatively low electrochemical potential (−2.71 V vs. SHE). Typically, SIBs share many chemical properties with LIBs (Kim et al., 2012; Li et al., 2013; Boyd and Augustyn, 2018; Zheng et al., 2019). The high concentrated WIS electrolytes produce ASIBs with better cycling stability. However, the easy crystallization of highly concentrated electrolytes at room temperature will seriously limit their practical application, and even damage the batteries (Wu et al., 2015; Reber et al., 2019; Zhang et al., 2020). Currently, the hydrate melts or bisalt, especially the adoption of asymmetric imide anions (such as FTFSI and PTFSI), are proved to be effective for reducing the viscosity and density as well as restraining crystallization by breaking the water structure and/or changing the probability of solvation structures with ion aggregations (Marcus, 2009; Brini et al., 2017; Suo et al., 2017), which thus results in the high solubility of salt anions (Suo et al., 2016; Zheng et al., 2019).

As reported in previous works, the commonly used salts in electrolytes of ASIBs are NaClO_4_, NaFSI, NaCF_3_SO_3_ (NaOTf), and NaTFSI due to their unique properties. Suo et al. first reported a Na^+^-conducting SEI layer on the surface of the NaTi_2_(PO_4_)_3_ anode in an electrolyte of 9.26 m sodium trifluoromethane sulfonate (NaCF_3_SO_3_ or NaOTf), which expands the electrochemical stability window of NaTi_2_(PO_4_)_3_ up to 2.5 V (vs. Na^+^/Na) (Suo et al., 2017). Kühnel and co-workers obtained an ultra-high-concentration (up to 37 M) sodium bis(fluorosulfonyl)imide (NaFSI) in water by rapid solidification of the entire supersaturated solution, offering a stable electrochemical window of 2.6 V. Strikingly, an aqueous NaTi_2_(PO_4_)_3_//Na_3_(VOPO_4_)_2_F sodium-ion battery with an electrolyte of 35 m NaFSI shows electrochemically reversible behaviors within an electrochemical window over 2.0 V (Kühnel et al., 2017). The NaFSI electrolytes with different concentrations are also shown to effectively broaden the voltage windows of ASIBs (Zheng et al., 2019). Another well-used electrolyte in ASIBs is NaClO_4_ solution. When its molality increases to 17 m, a stable electrochemical potential window of ~2.8 V can be realized (Nakamoto et al., 2017, 2018; Lee et al., 2019). However, the potential explosive risk and high toxicity may hinder the extensive use of NaClO_4_. Battaglia et al. explored a NaTi_2_(PO_4_)_3_//Na_3_(VOPO_4_)_2_F sodium-ion battery by employing the mixed NaFSI/NaFTFSI electrolyte (25 m NaFSI and 10 m NaFTFSI). The unique device demonstrates superb electrochemical performance in terms of cycling stability, reversible capacity, and energy density within a wide operating temperature range from −10 to 30°C, benefiting from the positive role of the mixed electrolyte (Reber et al., 2019). A new type of mixed WIS electrolytes containing inert cations (TEA^+^) is prepared by dissolving sodium trifluoromethanesulfonate (NaOTf) and tetraethylammonium trifluoromethanesulfonate (TEAOTf) in water. When the total salt concentration is up to 31 m (9 m NaOTf and 22 m TEAOTf), the unique NaOTf/TEAOTf electrolyte is endowed with a wide voltage window of ~3.3 V, and suppresses the dissolution of the positive transition metal as well (Jiang et al., 2019a).

### APIBS

Potassium-ion batteries (PIBs) are also considered to be a promising energy storage system due to their abundant potassium resources (Su et al., 2016; Eftekhari et al., 2017). Generally, potassium owns lower standard redox potential than its counterparts of Na and Li, which will guarantee PIBs with a potentially higher cell voltage. However, the higher ionization potential and larger ionic radius of K itself have limited the choice of electrode materials for advanced APIBs (Suo et al., 2017; Hwang et al., 2018). Thanks to the smaller Stokes radius of solvated K^+^ owing to its weak Lewis acidity, and the low interfacial reaction resistance due to the small desolvation activation energy, the K-containing electrolytes always display higher conductivity than its Li/Na counterparts (Komaba et al., 2015; Kim et al., 2017; Chen et al., 2020). Meanwhile, the weak oxidation resistance of electrolytes and the insufficient passivation on the surface of negative electrodes leads to modest reversible capacities, especially at the initial cycle, or in the high-voltage (>4.0 V) operation windows, which limits the huge development of APIBs (Hosaka et al., 2018). Therefore, it is essential to fit the high energy APIBs to purposefully explore suitable electrolytes.

Leonard and co-workers first reported the aqueous electrolyte of 30 m potassium acetate (KAc) for APIBs (Leonard et al., 2018). With the electrolyte, the KTi_2_(PO_4_)_3_ (KTP) anode displays good reversible behaviors within an extended electrochemical window from −1.7 to 1.5 V (vs. Ag/AgCl). Compared to the KAc and LiTFSI-based electrolytes with the same concentration, the bis(fluorosulfonyl)imide(KFSI)-based electrolytes exhibit higher conductivity (Chen et al., 2020). The electrolyte of 30 m KFSI also displays an electrochemical stability window from −1.55 to 2.42 V (vs. Hg/Hg_2_Cl_2_), which enables the *b*-perylene-3,4,9,10-tetracarboxylic dianhydride (*b*-PTCDA), and even *b*-PTCDA-based full batteries to stably operate in such high-concentration electrolytes without hydrogen evolution and material dissolution (Chen et al., 2020). Jiang et al. also investigated an aqueous 3,4,9,10-perylenetetracarboxylic diimide (anode)//K_1.85_Fe_0.33_Mn_0.67_[Fe(CN)_6_]_0.98_·0.77H_2_O (cathode) full device with 20 m KCF_3_SO_3_ (KOTf) WIS electrolyte, and the full battery exhibits an unprecedented performance in terms of reversible capacities and rate behaviors (Jiang et al., 2019b).

The asymmetric hydrate melts with an optimized eutectic system have been reported as a stable aqueous electrolyte with good fluidity and reduced viscosity/density, in which all water molecules participate in Li^+^ hydration shells (Yamada et al., 2016). With the introduced stable asymmetric anion (i.e., PTFSI^−^), the K(PTFSI)_0.12_(TFSI)_0.08_(OTf)_0.8_·2H_2_O as the alkali melts exhibits excellent water solubility and an expanded operating window of ~2.5 V (~2.14–4.65 V vs. K^+^/K), but does not suffer from the vulnerable S-F bond. Moreover, the ionic conductivity of the K(PTFSI)_0.12_(TFSI)_0.08_(OTf)_0.8_2H_2_O is maintained at ~34.6 mS cm^−1^, much higher than other typical non-aqueous electrolytes (~10 mS cm^−1^) (Zheng et al., 2019).

### AZIBS

Recently, AZIBs, due to their remarkable thermal stability, high theoretical specific capacity (~820 mAh g^−1^), intrinsic safety, and low cost of the Zn metal, are considered to be the most promising alternative to LIBs. Moreover, the metallic Zn is stable, and can be directly utilized as an electrochemically reversible anode in aqueous electrolytes (Zhang, 1996; Li et al., 2019). However, its extensive applications are still limited by suitable aqueous electrolytes with excellent thermal properties and safety. Previous works have shown that the alkaline aqueous electrolytes result in the formation of zinc dendrite and the by-product of ZnO, causing a poor cycle capacity and low CE values (Zhang et al., 2014; Wang et al., 2018a). Similarly, the Zn salts-based neutral or mildly acidic electrolytes with high concentrations are a very effective way to address these issues.

Typically, ZnSO_4_ and Zn(CF_3_SO_3_)_2_ solutions are commonly used electrolytes for AZIBs because of their excellent stability and compatibility (Song et al., 2018). Zhao et al. assembled a Zn//LiMn_0.8_Fe_0.2_PO_4_ aqueous hybrid-ion battery with 0.5 m ZnSO_4_ and 21 m LiTFSI as the WIS electrolyte. The unique device provides a high energy density of ~183 Wh kg^−1^ and a high operating voltage exceeding 1.8 V (Zhao et al., 2016). However, the ZnSO_4_ electrolyte for AZIBs always suffers from its intrinsically limited solubility and lower Zn stripping/plating efficiency. In contrast, the Zn(CF_3_SO_3_)_2_ electrolyte exhibits smaller polarization and higher CE values, which makes it suitable for wide application in aqueous ZIBs (Huang et al., 2019b; Xie et al., 2020). Mai's group designed a novel Zn//V_2_O_5_ aqueous hybrid-ion battery with the WIS electrolyte of 1 m Zn(CF_3_SO_3_)_2_ and 21 m LiTFSI. Compared to that with the Zn(CF_3_SO_3_)_2_ (1 m), the Zn//V_2_O_5_ battery with the WIS-electrolyte (21 m LiTFSI and 1 m Zn(CF_3_SO_3_)_2_) displayed a more stable charge/discharge plateau and cycling performance (Hu et al., 2017). Furthermore, owing to the large-size TFSI^−^ anions, the Zn(TFSI)_2_, as a novel organic zinc salt can effectively reduce the solvation effect. Wang's group developed a WIS electrolyte of 1 m Zn(TFSI)_2_ + 20 m LiTFSI, which is capable of retaining the water in an open atmosphere. It effectively promotes the dendrite-free plating/stripping of metallic Zn with nearly 100% CE and brings unprecedented reversibility to the aqueous ZIBs with either LiMn_2_O_4_ or O_2_ cathodes (Wang et al., 2018a). Additionally, a new low-cost WIS electrolyte of 30 m ZnCl_2_ can deliver a wide electrochemical window of 2.3 V due to its fewer side reactions and low oxidative Cl^−^ (Zhang et al., 2018). In the symmetric Zn||Zn cell with a 30 m ZnCl_2_ electrolyte, the Zn electrode renders a high CE of 95.4% and a high stable galvanostatic charge-discharge profile of over 600 h without any significant overpotential fluctuation (Zhang et al., 2018).

### AMIBS

Multivalent ions, as good transporters, can carry more electrons than monovalent ions. Besides the Zn^2+^, another bivalent metal ion of Mg^2+^ with low reduction potential (−2.37 V) is also considered as a predominant charge carrier for AMIBs due to the high volumetric specific capacity of Mg (~3833 mAh L^−1^) and the total lakc of dendrite growth (Rasul et al., 2012; Song et al., 2015; Xu et al., 2015; Sun et al., 2016). The current development and practical applications of the electrolytes for AMIBs are limited by the corrosion of the electrolytes (Wang et al., 2017; Zhao et al., 2020). The electrochemical Mg dissolution occurs at a high overpotential which restrains the selection of solvents (Hebié et al., 2017). Consequently, the commonly used anions (Cl^−^, ${\text{SO}}_{4}^{2-}$, ${\text{ClO}}_{4}^{-}$, CF_3_${\text{SO}}_{3}^{-}$) in other ARMIBs cannot be directly applied to AMIBs. Moreover, the high charge density of multivalent ions will induce strong coulombic interactions with both the lattice of electrolyte solvents and electrode materials, which is an adverse factor for improving electrochemical performance (Lapidus et al., 2014; Wang et al., 2018b). So far, the AMIBs are still in its infancy; only a few possible materials based on WIS electrolytes show reversible performance toward AMIBs.

Moreover, Mg(TFSI)_2_, as a neutral molecule, is completely non-corrosive, safe, and green, and can be anticipated for AMIBs application (Yoo et al., 2013). Wang et al. fabricated a poly pyromellitic dianhydride//Li_3_V_2_(PO_4_)_3_ device by using 4 m Mg(TFSI)_2_ as the electrolyte. The full cell exhibits superior electrochemical properties including an excellent rate capability, high power density, and high capacity retention in an electrochemical window of 1.9 V (Wang et al., 2017).

### AAIBS

Aluminum is the first abundant metal element in the earth's crust, which has been investigated widely as the anode material for AAIBs (Wang et al., 2016). Moreover, the ion radius of Al^3+^ (0.054 nm) is much smaller than Li^+^ (0.076 nm), which ensures the rapid insertion/extraction of Al^3+^ during the charge/discharge process (Rudd and Gibbons, 1994; Li et al., 2007; Das et al., 2017; Yang et al., 2019b). Furthermore, the Al anode possesses a large gravimetric/volumetric capacity (~2,980 mAh g^−1^/~8,046 mAh cm^−3^), due to its unique three-electron transfer capability. However, the low ionic conductivity, corrosion with low concentration electrolytes, and dendrites growth still limits the electrochemical stability of electrolytes, thereby limiting its large-scale energy applications (Liu et al., 2012; Nakayama et al., 2015; Zhao et al., 2018). So, the key for developing AAIBs is to exploit WIS electrolytes toward high-performance electrodes, which can enable the dissolution of dendrites, thereby significantly improve the cycling stability.

AlCl_3_, due to the ultra-low cost and safety, is widely used as the electrolytes for AAIBs. Pan et al. reported a high-concentration aqueous AlCl_3_ solution (3.382 m) as the electrolyte for a novel Al/AlCl_3_/graphite aqueous battery, which can stably operate within an electrochemical stability window of ~4 V, and exhibit a large capacity of ~165 at 500 mA g^−1^ along with a high CE of 95% over 1,000 cycles (Pan et al., 2019). Additionally, the Al(OTf)_3_ is another type of electrolyte with a noncorrosive property, which makes it more favorable than corrosive AlCl_3_ when applied in AAIBs (Das et al., 2017). Chen's group introduced the electrolyte of 5 m Al(OTf)_3_ for electrochemical evaluation of the Prussian Blue Analogs-type FeFe(CN)_6_ (FF-PBA) cathode. Appealingly, with the electrolyte, the FF-PBA shows an extraordinarily high initial discharge capacity of ~116 mAh g^−1^ and long cycle life with a capacity fading of 0.39% per cycle in the expanded operation window of 2.65 V (Zhou et al., 2019). Until now, there are few other WIS electrolytes reported for AAIBs.

## Conclusion

Electrolytes, as ionic transport intermediates with inherent ionic conductivity, mobility, interfacial characteristics, and other properties, play a critical role in enhancing the cycle performances, rate capacity, and safety of batteries. WIS electrolytes with highly concentrated salt solutions specifically can expand electrochemical potential windows of aqueous devices up to about 3 V and result in low solvent (water molecules) activities and high chemical stabilities (restraining side reactions). Moreover, the formation of stable SEI film also endows the cells with a high energy density and excellent cycling stability. This mini review mainly focuses on the WIS electrolytes for ARMIBs and summarizes the recent investigation of WIS electrolytes in non-lithium monovalent (Na, K) and multivalent (Zn, Mg, Al) ion batteries.

However, research based on the WIS electrolytes is still at the primary stage, according to the achievements reported so far; the challenges and prospects for the future development of WIS electrolytes toward non-lithium ARMIBs are proposed as follows. First, the scientific foundation for the highly concentrated WIS electrolytes will render some novel yet unknown concepts, which may be in conflict with current classic theories, and should be further unveiled with the elegant combination of *in/ex-situ* spectroscopic techniques and theoretical simulation/calculation. For instance, an in-depth understanding of intrinsic ionic transport and functional SEI formation mechanisms in the WIS electrolyte, which are distinct from the conventional SIW systems, should be comprehensively conducted. Second, further exploration of appropriate salts, particularly with high thermodynamic stability, super ionic conductivity, and good compatibility with both electrodes at a low cost, should also be taken into account for high-performance WIS electrolytes, considering their practical commercial applications. Third, the balance between high-concentration electrolytes and low viscosity and crystallization also needs to be solved.

Despite facing huge difficulties and challenges, we firmly believe that aqueous rechargeable batteries based on WIS electrolytes will receive rapid and sustained development in the near future. This will result in new avenues for the future energy storage landscape.

## Author Contributions

YW: formal analysis, investigation, writing—original draft, and writing—review & editing. XM: investigation. JS: formal analysis and writing—review & editing. YL: visualization and software. LH: formal analysis, investigation, conceptualization, writing—review & editing, and funding acquisition. All authors contributed to the article and approved the submitted version.

## Conflict of Interest

The authors declare that the research was conducted in the absence of any commercial or financial relationships that could be construed as a potential conflict of interest.
